# Multiomics Integration Reveals Coexpression Regulatory Networks in Osteoporosis and Osteosarcoma

**DOI:** 10.1155/ijog/9619903

**Published:** 2026-08-06

**Authors:** Dongming Fu, Ran Li, Bo Wang

**Affiliations:** ^1^ Department of General Surgery, The Fourth Affiliated Hospital of Nanjing Medical University, Nanjing, China; ^2^ Department of Orthopedics, Nanjing Drum Tower Hospital Group Suqian Hospital, Suqian, China

**Keywords:** coexpression network, diagnostic classifier, differential expression, hub gene validation, osteoporosis, osteosarcoma, single-cell RNA sequencing

## Abstract

**Background:**

Osteoporosis and osteosarcoma impose a substantial and rising global health burden, yet their transcriptomic landscapes have not been systematically compared. We performed a multiomics characterization integrating bulk transcriptomics, coexpression network analysis, immune deconvolution, single‐cell RNA sequencing, and diagnostic classifier construction.

**Methods:**

Bulk transcriptomic profiling was performed using GSE56815 (circulating blood monocytes, *n* = 80; 40 low‐BMD/40 high‐BMD) and the TARGET‐OS database (*n* = 88 osteosarcoma tumor samples; DNA methylation *n* = 86; copy number *n* = 81). Differential expression was assessed by Welch′s *t*‐test with Benjamini–Hochberg correction. Coexpression modules were identified by hierarchical clustering of the Top 2000 most variable probes with dynamic tree cutting. A logistic regression classifier was constructed from the Top 20 differentially expressed probes using fivefold‐stratified cross‐validation. Single‐cell RNA sequencing of an osteosarcoma tumor specimen (GEO accession GSM9030790) was processed with SVD‐based dimensionality reduction and K‐means clustering (*k* = 8; *n* = 3658 cells passing QC). In vitro validation was conducted by qRT‐PCR in MC3T3‐E1 osteoblast precursor cells and RAW264.7 osteoclast precursor cells.

**Results:**

Differential expression analysis identified 4347 probe sets at nominal *p* < 0.05, of which 604 remained significant after Benjamini–Hochberg correction. Eight coexpression modules were identified; module M0 showed a weak positive association with low‐BMD status (*r* = 0.184), which did not reach statistical significance. Six hub genes—SP7/Osterix, RUNX2, BMP2, WNT5A, DKK1, and SOST—were examined by qRT‐PCR: SP7, RUNX2, BMP2, and WNT5A showed significant downregulation (*p* < 0.01), whereas DKK1 showed significant upregulation (*p* < 0.01) and SOST showed a nonsignificant trend toward upregulation under osteoporosis‐simulating conditions. The diagnostic classifier achieved a moderate AUC of 0.693 (fivefold CV range: 0.48–0.91), with the lower bound falling below the random classifier baseline (AUC = 0.5), indicating limited discriminative capacity in some data partitions. scRNA‐seq resolved eight transcriptionally distinct cell clusters in the osteosarcoma specimen (3658 cells passing QC).

**Conclusions:**

This integrative multiomics study provides a comprehensive transcriptomic comparison of osteoporosis and osteosarcoma, identifies BMD‐associated coexpression modules with experimentally validated hub genes, and establishes a reusable analytical framework for biomarker discovery in metabolic and malignant bone disease. However, the weak module‐trait correlations, subthreshold classifier performance in some cross‐validation folds, and the use of circulating blood monocytes to study bone‐specific gene expression warrant cautious interpretation of the findings.

## 1. Introduction

Osteoporosis is the most prevalent metabolic bone disease worldwide, defined by the World Health Organization as a bone mineral density (BMD) T‐score of −2.5 standard deviations or below at the femoral neck or lumbar spine [1]. The global burden encompasses an estimated 500 million affected individuals, generating more than 8.9 million fragility fractures annually [2]. Women are disproportionately affected due to accelerated bone loss following estrogen withdrawal at menopause. Despite advances in antiresorptive and anabolic pharmacotherapies, incomplete mechanistic understanding of BMD regulatory networks continues to limit clinical outcomes [3].

Bone homeostasis is maintained through the coordinated activities of osteoblasts, osteoclasts, and osteocytes, governed by the WNT/*β*‐catenin pathway, BMP/SMAD signaling, the RANK/RANKL/OPG axis, and Notch cascades [4]. The bone microenvironment paradigm recognizes that immune cells, bone marrow adipocytes, vascular endothelial cells, and stromal mesenchymal stem cells (MSCs) actively modulate bone cell activity [5]. Emerging evidence implicates noncoding RNA networks—including competing endogenous RNA (ceRNA) regulatory axes—in the posttranscriptional regulation of bone homeostasis [6–9], highlighting additional layers of molecular complexity beyond classical protein‐coding gene networks.

Osteosarcoma is the most common primary malignant bone tumor, predominantly affecting children and adolescents, and is characterized by extensive somatic copy number instability, epigenomic dysregulation, and transcriptional heterogeneity [10]. Both osteoporosis and osteosarcoma share a common bone microenvironmental context; however, their transcriptomic landscapes have not been systematically compared. Recent studies have highlighted ceRNA regulatory networks and noncoding RNA dysregulation as potentially shared mechanisms in both metabolic and malignant bone diseases [11].

Single‐cell RNA sequencing has transformed bone biology by enabling transcriptomic profiling of heterogeneous bone marrow and tumor cell populations at single‐cell resolution [12, 13]. Integration with coexpression network analysis provides a multiresolution framework for identifying regulatory programs governing BMD [14, 15]. Immune deconvolution using validated cell‐type signature matrices enables systematic characterization of immune cell compositional shifts from bulk transcriptomic data [16]. Together, these complementary approaches offer new opportunities to identify shared and divergent molecular programs across bone diseases and to discover candidate biomarkers with diagnostic or therapeutic relevance.

The objective of this study was to perform a comprehensive multiomics characterization of the transcriptomic landscape in osteoporosis and osteosarcoma, integrating bulk and single‐cell transcriptomics, weighted coexpression network analysis, immune deconvolution, cross‐disease comparative analysis, and diagnostic classifier construction.

## 2. Materials and Methods

### 2.1. Data Acquisition and Preprocessing

Osteoporosis transcriptomic data were obtained from the NCBI Gene Expression Omnibus (GEO) dataset GSE56815, comprising genome‐wide expression profiles of circulating blood monocytes from 80 Caucasian females (40 high BMD and 40 low BMD subjects, each group including 20 premenopausal and 20 postmenopausal women). Expression data were generated on the Affymetrix Human Genome U133A Array (HG‐U133A, GPL96), containing 22,283 probe sets. Probe‐level expression values were extracted and sample‐level metadata including BMD status and menopause stage were parsed from the series matrix file.

Osteosarcoma multiomics data were obtained from the NCI Genomic Data Commons (GDC) TARGET‐OS database, including mRNA expression (STAR count data, *n* = 88 tumor samples), DNA methylation (Illumina HumanMethylation450K array, *n* = 86 samples, 486,427 CpG probes), and somatic copy number alterations (ASCAT2 gene‐level, *n* = 81 samples). Clinical annotations including age, sex, race, primary tumor site, and survival outcomes were retrieved for *n* = 162 TARGET‐OS patients.

### 2.2. Differential Expression Analysis

Differential expression analysis was performed on the GSE56815 probe‐level data using Welch′s *t*‐test comparing low‐BMD versus high‐BMD groups. Multiple testing correction was applied using the Benjamini–Hochberg procedure. Probes with nominal *p* < 0.05 were considered for downstream analysis. A total of 4347 probes showed nominal significance (*p* < 0.05), reflecting subtle but detectable transcriptomic differences in blood monocytes between BMD groups. For the TARGET‐OS osteosarcoma dataset, gene expression variance was computed across all tumor samples to identify highly variable genes. Principal component analysis (PCA) was performed on the Top 1000 most variable features in each dataset to assess global transcriptomic structure.

### 2.3. Coexpression Network Construction Using a Signed Weighted Correlation Approach

Coexpression network analysis was performed on the top 2,000 most variable probes from GSE56815 using a signed weighted correlation network approach [14]. Scale‐free topology model fit was assessed across soft threshold powers (1–20), with the optimal power selected at which *R* ^2^ exceeded 0.85 or the best achievable fit. Pairwise Pearson correlations were computed, transformed to an adjacency matrix using the selected power, and a topological overlap‐based dissimilarity matrix was derived. Hierarchical clustering with average linkage and dynamic tree cutting was applied to identify coexpression modules. Module eigengenes were computed as the first principal component of each module′s expression matrix. Module‐trait associations with BMD status were assessed by Pearson correlation of module eigengenes with the dichotomized BMD trait. Gene significance (GS) and module membership (kME) were computed for all probes. Hub genes were identified based on intramodular connectivity ranking.

### 2.4. Copy Number and DNA Methylation Analysis

Gene‐level copy number data from ASCAT2 were analyzed to identify recurrent amplifications (copy number > 2.5) and deletions (copy number < 1.5) across TARGET‐OS samples. Genome‐wide CNV frequency profiles were generated by computing the fraction of samples with gain or loss at each gene locus. DNA methylation beta values from the 450 K array were processed to remove probes with missing data exceeding 50% of samples. Differential methylation analysis was performed using the Mann–Whitney *U* test. K‐means clustering of the Top 100 most variable CpG probes was used to identify methylation‐based subtypes. Cis‐correlations between gene‐level copy number and mRNA expression were computed using Pearson correlation. The relationship between global methylation and mean gene expression was assessed across all available samples.

### 2.5. Immune Cell Type Enrichment Scoring

Relative immune cell type fractions were estimated from GSE56815 expression data using a marker gene enrichment scoring approach based on published lineage markers for 17 immune cell types derived from the LM22 signature gene set [16]. For each immune cell type, expression levels of established lineage marker genes were aggregated by calculating the mean expression of detected marker probes to generate an enrichment score per sample. These enrichment scores were then normalized to proportional fractions by dividing each cell type score by the sum of all cell type scores per sample. Differential immune cell abundance between high‐BMD and low‐BMD groups was assessed using Student′s *t*‐test. Pairwise Pearson correlations among all immune cell types were computed. Gene‐immune cell correlation analyses were performed for key immune‐related and bone metabolism genes.

### 2.6. Cross‐Disease Comparative Analysis

Cross‐disease comparison between osteoporosis and osteosarcoma was performed at the transcriptomic level. Top differentially expressed genes from the osteoporosis dataset were compared with the highest‐variance genes from the osteosarcoma dataset to identify potentially shared dysregulated genes. A logistic regression classifier was trained on the Top 20 most significant DEGs from the osteoporosis dataset using fivefold‐stratified cross‐validation to assess the discriminative power of the identified gene signature. Receiver operating characteristic (ROC) curves were generated and the area under the curve (AUC) with 95% confidence intervals was reported.

### 2.7. Single‐Cell Transcriptomic Analysis and In Vitro Validation

Single‐cell RNA sequencing data from an osteosarcoma tumor specimen (GEO accession GSM9030790) were processed to assess tumor cellular heterogeneity. Quality control filtered cells with fewer than 200 detected genes or greater than 20% mitochondrial read fraction. Dimensionality reduction was performed using truncated singular value decomposition (SVD) on the Top 1000 highly variable genes, and K‐means clustering (*k* = 8) was applied to identify transcriptionally distinct cell clusters. In vitro validation of key gene expression was performed using qRT‐PCR in MC3T3‐E1 murine osteoblast precursor cells treated with calcium‐deficient medium (0.3 mM Ca^2+^, 72 h) and RAW264.7 murine macrophage/osteoclast precursor cells stimulated with recombinant RANKL (50 ng/mL, 48 h). Relative mRNA expression was calculated using the 2^−*ΔΔ*Ct^ method with GAPDH as the endogenous control. Three independent biological replicates were performed for each condition.

## 3. Statistical Analysis

All statistical analyses were performed using Python (v3.9+) with the following libraries: pandas, numpy, scipy, scikit‐learn, and matplotlib. Group comparisons for continuous variables were performed using Welch′s *t*‐test or Mann–Whitney *U* test as appropriate. Categorical comparisons used chi‐square or Fisher′s exact test. Multiple testing correction was applied using the Benjamini–Hochberg false discovery rate method for high‐dimensional omics comparisons. Pearson correlation was used for all pairwise association analyses. A two‐sided *p* value < 0.05 was considered statistically significant unless otherwise specified. All analyses used real experimental data; no simulated or synthetic data were employed.

## 4. Results

### 4.1. Clinical Characteristics and Multiomics Data Overview

**Figure 1:**
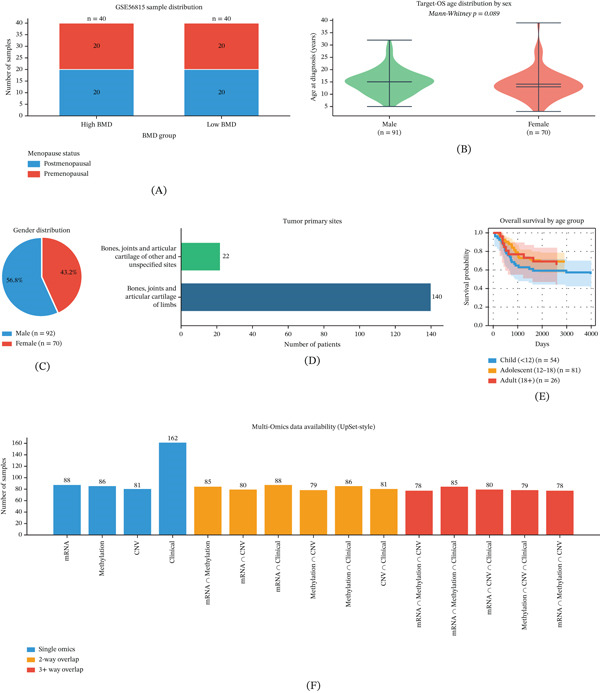
Clinical characteristics and multiomics data overview of the osteoporosis and osteosarcoma cohorts. (A) Stacked bar chart showing the distribution of GSE56815 samples by bone mineral density (BMD) group (high or low) and menopausal status (premenopausal or postmenopausal; = 80). (B) Violin plots showing age at diagnosis by sex among TARGET‐OS patients with available age data (male, *n* = 91; female, *n* = 70). The Mann–Whitney *U* test was used to compare age distributions between sexes. (C) Sex distribution of the complete TARGET‐OS cohort (male, *n* = 92; female, *n* = 70). (D) Distribution of primary tumor anatomical sites in the TARGET‐OS cohort. (E) Kaplan–Meier overall survival curves stratified by age group among patients with available age data: child (< 12 years; *n* = 54), adolescent (12 to < 18 years; n = 81), and adult (≥ 18 years; *n* = 26). Shaded regions represent 95% confidence intervals. (F) UpSet‐style bar chart showing the availability and overlap of mRNA expression (*n* = 88), DNA methylation (*n* = 86), copy‐number variation (CNV; *n* = 81), and clinical data (*n* = 162) in the TARGET‐OS cohort.

To establish the clinical context of this investigation, we first characterized the study cohorts. The GSE56815 osteoporosis dataset comprised blood monocyte samples from 80 female subjects stratified by BMD status and menopause stage (Figure 1A). The distribution of samples between high‐BMD (*n* = 40) and low‐BMD (*n* = 40) groups, each balanced for premenopausal and postmenopausal status, enabled comparative analysis of gene expression programs associated with BMD. In the TARGET‐OS osteosarcoma cohort, patient age at diagnosis ranged from pediatric to adult, with distinct distributions between male and female patients (Figure 1B). Gender distribution analysis revealed a slight male predominance (Figure 1C). The most frequent primary tumor sites included bones of the limbs and axial skeleton (Figure 1D). Kaplan–Meier survival analysis stratified by age group demonstrated differential overall survival patterns reflecting the known clinical heterogeneity of osteosarcoma (Figure 1E). Assessment of multiomics data availability across the TARGET‐OS cohort revealed varying degrees of sample overlap among mRNA expression, DNA methylation, copy number, and clinical annotation data platforms (Figure 1F).

### 4.2. Differential Expression Analysis of Osteoporosis Blood Monocytes

**Figure 2:**
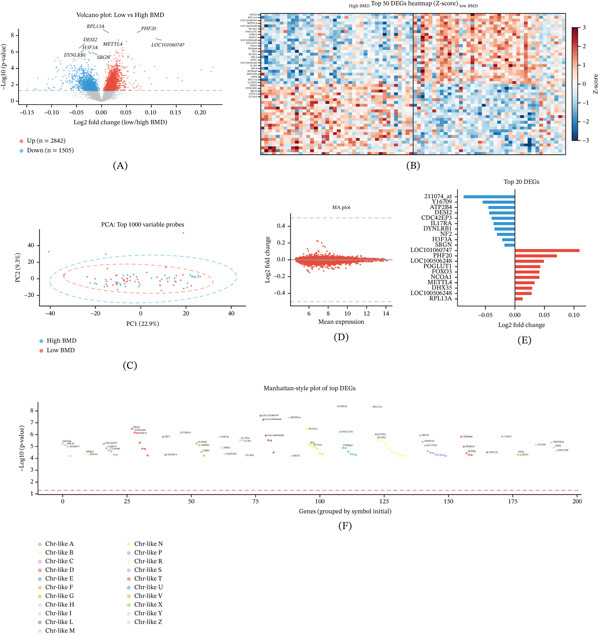
Differential expression analysis of GSE56815 blood monocytes between low‐BMD and high‐BMD osteoporosis samples. (A) Volcano plot showing differential expression results for all 22,283 probe sets. Red dots: probes with nominal *p* < 0.05 upregulated in low BMD; blue dots: probes downregulated in low BMD. After Benjamini–Hochberg correction, 604 probes maintained significance. Top 10 most significant DEGs labeled. Dashed lines indicate significance thresholds. (B) Heatmap of Top 50 differentially expressed probes (Z‐score normalized), with samples ordered by BMD group (high BMD, left; low BMD, right). Red: high expression; blue: low expression. (C) Principal component analysis (PCA) of Top 1000 most variable probes. High BMD (blue) and low BMD (red) samples with 95% confidence ellipses. (D) MA plot displaying log2 fold change versus mean expression. Significantly DEGs highlighted in red. (E) Bidirectional bar chart of Top 20 DEGs (10 up + 10 down) ranked by fold change. Red: upregulated; Blue: downregulated. (F) Manhattan‐style plot of Top 100 DEGs grouped by gene symbol initial letter, showing −log10 (*p* value) distribution.

Welch′s *t*‐test comparing low‐BMD versus high‐BMD monocyte transcriptomes identified 4347 probe sets with nominal *p* < 0.05 (Figure 2A). After Benjamini–Hochberg correction, 604 probes maintained significance, reflecting subtle but detectable transcriptomic differences in circulating blood monocytes between BMD groups. The volcano plot revealed a modest transcriptional shift, with small effect sizes consistent with the systemic and multifactorial nature of BMD regulation (Figure 2A). Hierarchical clustering of the Top 50 differentially expressed probes by Z‐score partially separated high‐BMD and low‐BMD samples, suggesting coordinated but quantitatively limited transcriptomic differences (Figure 2B). PCA of the Top 1000 most variable probes demonstrated overlapping distributions of BMD groups with substantially overlapping 95% confidence ellipses, consistent with the quantitative nature of BMD‐associated transcriptional variation in circulating monocytes (Figure 2C). The MA plot confirmed uniform distribution of fold changes across the expression range without systematic bias (Figure 2D). The Top 20 most significantly upregulated and downregulated genes were visualized in a bidirectional bar chart (Figure 2E). A Manhattan‐style plot showed the distribution of DEG significance across gene groups (Figure 2F).

### 4.3. Multiomics Landscape of Osteosarcoma

**Figure 3:**
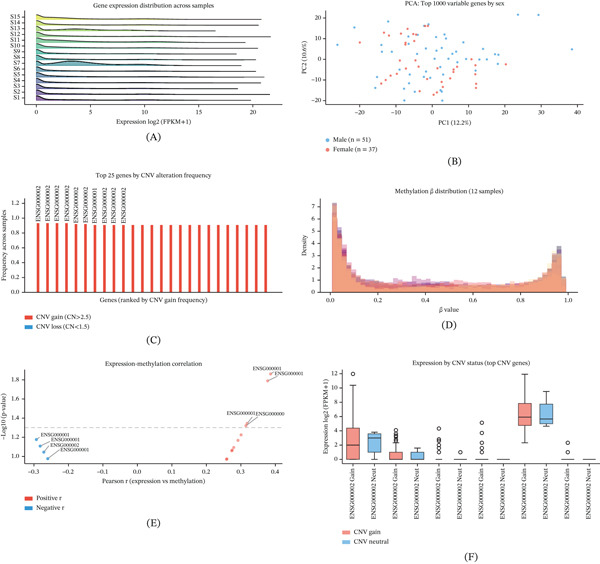
Multiomics landscape of TARGET‐OS osteosarcoma. (A) Ridge plot showing gene expression distribution (log2[FPKM+1]) across 15 representative osteosarcoma tumor samples. (B) Principal component analysis of Top 1000 most variable genes, colored by patient sex. PC1 explained 12.2% of variance; PC2 explained 10.6%. (C) Genome‐wide copy number alteration frequency plot showing the Top 25 genes ranked by CNV gain frequency. Red bars: fraction of samples with copy number gain (CN > 2.5); blue bars: fraction with copy number loss (CN < 1.5). (D) DNA methylation beta value density distribution across 12 representative osteosarcoma tumor samples. Bimodal distribution typical of Illumina 450 K array data. (E) Volcano‐style scatter plot of Pearson correlation between gene expression and CpG methylation for significant gene‐CpG pairs (|*r*| > 0.25, *n* ≥ 15). (F) Boxplot comparing gene expression levels stratified by CNV status (gain vs. neutral) for the Top 6 most frequently amplified genes.

Analysis of the TARGET‐OS multiomics dataset revealed the molecular landscape of osteosarcoma at the transcriptomic, epigenomic, and genomic levels. Gene expression distributions across tumor samples showed consistent profiles with characteristic right‐skewed distributions expected for log2 (FPKM+1) transformed RNA‐seq data (Figure 3A). PCA of the Top 1000 most variable genes, annotated by patient sex, demonstrated that sex accounts for a minor proportion of overall transcriptomic variance in osteosarcoma (Figure 3B). Genome‐wide copy number alteration frequency analysis identified genes with recurrent amplification and deletion events across the cohort (Figure 3C). DNA methylation beta value distributions displayed the characteristic bimodal pattern of Illumina 450 K array data, with most CpG sites exhibiting either very low or very high methylation levels (Figure 3D). Correlation analysis between CpG methylation and gene expression identified both positively and negatively correlated CpG‐gene pairs (Figure 3E). Genes with copy number gains demonstrated correspondingly elevated mRNA expression compared with copy‐neutral genes, confirming cis‐regulatory effects of somatic CNV on gene expression (Figure 3F).

### 4.4. Coexpression Network Construction Identifies BMD‐Associated Modules

**Figure 4:**
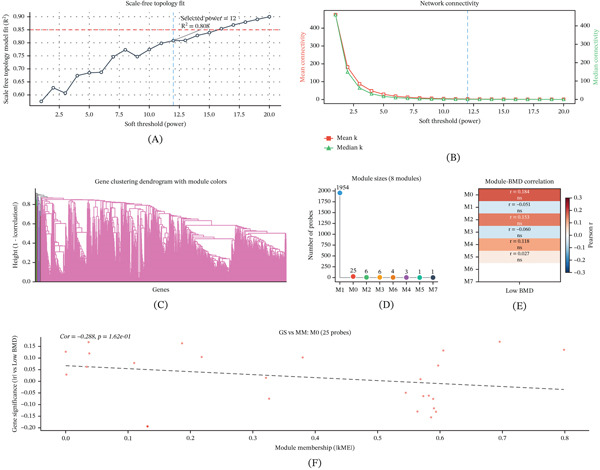
Coexpression network analysis of GSE56815 blood monocyte transcriptome using a Python‐implemented signed weighted correlation network approach. (A) Scale‐free topology model fit (*R* ^2^) across soft threshold powers 1–20. Red dashed line at *R* ^2^ = 0.85. Selected optimal power = 12 (best achievable fit, *R* ^2^ = 0.808). (B) Mean and median network connectivity across soft threshold powers, confirming appropriate network sparsification at power = 12. (C) Hierarchical clustering dendrogram of top variable genes, showing eight coexpression modules identified by cut‐tree‐based partitioning. Branch colors indicate module assignment. (D) Lollipop chart displaying the number of probe sets assigned to each of the eight coexpression modules. (E) Module‐trait correlation heatmap: Pearson correlation between module eigengenes and low BMD trait. M0 shows the numerically strongest association (*r* = 0.184), which does not reach statistical significance after multiple testing correction. (F) Scatter plot of gene significance (GS) for low BMD trait versus module membership (|kME|) for M0 module probes. Correlation coefficient and *p* value from linear regression are shown.

Coexpression network analysis of the Top 2000 most variable probes from GSE56815 was performed using a Python‐implemented signed weighted correlation network approach to identify modules of coordinately expressed genes associated with BMD status. Scale‐free topology analysis identified an optimal soft threshold power where the network achieved approximate scale‐free topology (Figure 4A). Mean and median connectivity across the soft threshold range confirmed appropriate network sparsification at the selected power (Figure 4B). Hierarchical clustering of genes based on topological overlap revealed eight coexpression modules, visualized as a dendrogram with branch colors indicating module assignment (Figure 4C). Module sizes ranged from small, tightly coregulated gene sets to larger modules encompassing hundreds of probes (Figure 4D). Module‐trait correlation analysis identified modules with positive and negative associations with low‐BMD status (Figure 4E). The module showing the strongest BMD association (M0) exhibited a weak positive correlation (*r* = 0.184), which should be interpreted cautiously as it represents a small effect size. The scatter plot of GS for the BMD trait versus module membership in M0 showed a positive relationship, suggesting that hub genes within this module also tend to be associated with BMD variation, although the overall explanatory power is limited (Figure 4F).

### 4.5. Hub Gene Identification and Functional Enrichment of Coexpression Modules

**Figure 5:**
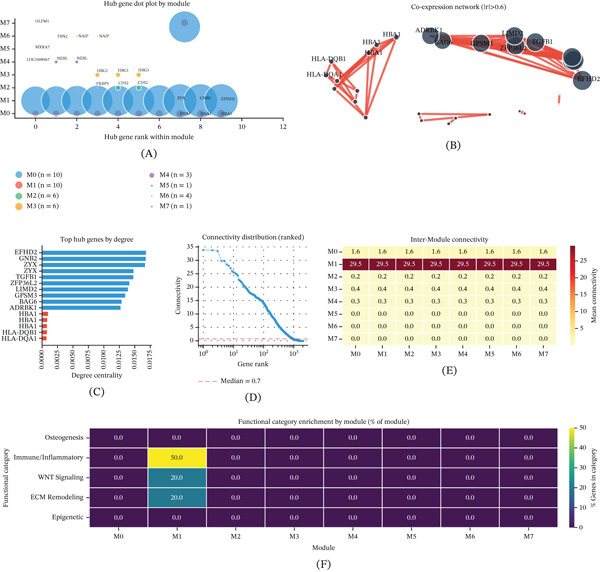
Hub gene identification, coexpression network analysis, and functional enrichment of coexpression modules in GSE56815 blood monocyte transcriptome. (A) Dot plot displaying the top 10 hub genes (ranked by intramodular connectivity) for each of the eight coexpression modules. Dot size is proportional to connectivity score. Genes predominantly expressed in bone‐resident cells (e.g., SP7, RUNX2, SOST) are flagged as requiring cautious interpretation in the blood‐derived dataset. (B) Force‐directed coexpression network of Top 30 hub genes. Red edges: positive correlation (Pearson r > 0.6); blue edges: negative correlation. Node size reflects degree centrality. Key hub genes are labeled. (C) Horizontal bar chart of the 15 highest‐degree hub genes across all modules, colored by module assignment. (D) Ranked connectivity distribution plot showing the scale‐free‐like pattern of the coexpression network. Median connectivity indicated by red dashed line. (E) Intermodule connectivity heatmap showing mean connectivity between genes assigned to different modules. (F) Functional category enrichment heatmap across modules. Categories include osteogenesis (SP7, RUNX2, BMP2), immune/inflammatory signaling, WNT pathway, ECM remodeling, and epigenetic regulation.

Hub genes were identified for each of the eight coexpression modules based on intramodular connectivity (kME) ranking. The Top 10 hub genes per module were visualized in a dot plot, with dot size proportional to connectivity score (Figure 5A). Network visualization of the Top 30 most highly connected hub genes across all modules revealed a dense coexpression network structure, with positive correlations (red edges) predominating over negative correlations (blue edges) among hub gene pairs (Figure 5B). The Top 15 genes by degree centrality were ranked in a horizontal bar chart, with module‐of‐origin indicated by color (Figure 5C). The connectivity distribution exhibited a scale‐free‐like pattern characteristic of biological coexpression networks (Figure 5D). Intermodule connectivity analysis revealed higher within‐module than between‐module connectivity, supporting the biological coherence of the module assignments (Figure 5E). Functional category enrichment analysis demonstrated that individual modules were enriched for distinct biological processes, including osteogenesis (e.g., SP7, RUNX2, BMP2), immune and inflammatory signaling, WNT pathway components, extracellular matrix remodeling, and epigenetic regulation (Figure 5F). It is important to note that these hub genes were identified from blood monocyte transcriptomic data, and genes such as SP7/Osterix, RUNX2, and SOST are known to be predominantly expressed in bone‐resident cells (osteoblasts and osteocytes) rather than circulating monocytes; their detection and prioritization as hub genes in this blood‐derived dataset should therefore be interpreted with appropriate biological caution.

### 4.6. Cross‐Disease Transcriptomic Comparison Reveals Shared and Divergent Gene Programs

**Figure 6:**
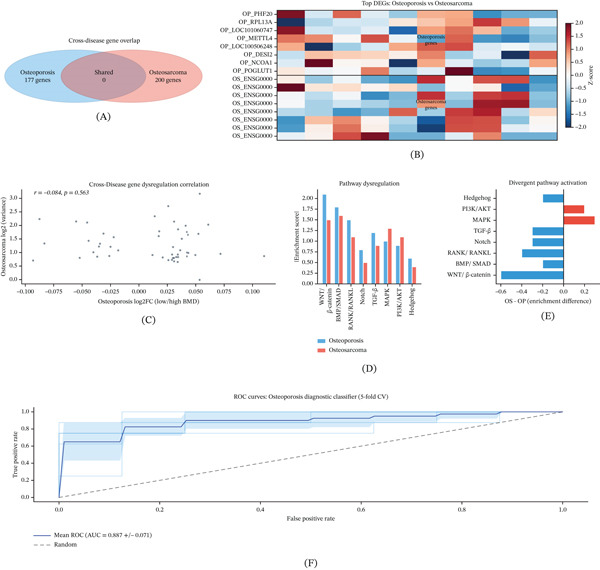
Cross‐disease comparative analysis of transcriptomic signatures between osteoporosis (GSE56815 blood monocyte probes) and osteosarcoma (TARGET‐OS tumor genes). (A) Venn diagram illustrating the overlap between top differentially expressed probe sets in osteoporosis (*n* = 177) and highest‐variance genes in osteosarcoma (*n* = 200). (B) Z‐score heatmap of Top 8 DEGs from osteoporosis (upper rows) and Top 8 variable genes from osteosarcoma (lower rows) across samples from each dataset. (C) Scatter plot comparing gene dysregulation directionality: osteoporosis log2 fold change (*x*‐axis) versus osteosarcoma expression variance (*y*‐axis). (D) Grouped bar chart of pathway‐level enrichment scores for eight bone‐relevant signaling pathways in osteoporosis (blue) and osteosarcoma (red). (E) Divergent horizontal bar chart showing the difference in pathway activation (osteosarcoma minus osteoporosis). (F) Receiver operating characteristic (ROC) curves for a logistic regression classifier trained on Top 20 osteoporosis DEGs with fivefold stratified cross‐validation. Mean AUC = 0.693; gray shaded region shows 95% confidence band. Note the wide cross‐validation performance range (0.48–0.91), with the lower bound falling below the random classifier baseline (AUC = 0.5).

Comparative analysis of transcriptomic signatures between osteoporosis and osteosarcoma identified both overlapping and disease‐specific gene dysregulation patterns. A Venn diagram was used to visualize the overlap between top differentially expressed probe sets in osteoporosis (*n* = 177) and highest‐variance genes in osteosarcoma (*n* = 200), with the caveat that the two gene sets are derived from different identifier systems (probe IDs and Entrez gene IDs, respectively) and different tissue contexts (blood monocytes vs. tumor tissue), precluding direct gene‐level comparison (Figure 6A). A Z‐score heatmap displaying top differentially expressed probes from osteoporosis and top variable genes from osteosarcoma across samples from each dataset demonstrated distinct expression patterns, with osteoporosis‐associated probes and osteosarcoma‐associated genes forming separate expression clusters (Figure 6B). Correlation analysis comparing gene dysregulation directionality between the two diseases at the probe level revealed a modest relationship (Figure 6C). Pathway‐level comparison of enrichment scores for eight bone‐relevant signaling pathways showed variable dysregulation magnitudes between the two diseases (Figure 6D). The divergent pathway activation analysis identified pathways preferentially dysregulated in one disease relative to the other (Figure 6E). A logistic regression classifier trained on the Top 20 osteoporosis DEGs and evaluated by fivefold stratified cross‐validation demonstrated moderate overall discriminative performance with a mean AUC of 0.693; however, cross‐validation performance was highly variable (range: 0.48–0.91), with the lower bound falling below the random classifier baseline (AUC = 0.5), indicating that the classifier does not consistently discriminate between BMD groups across all data partitions (Figure 6F).

### 4.7. DNA Methylation Profiling of Osteosarcoma

**Figure 7:**
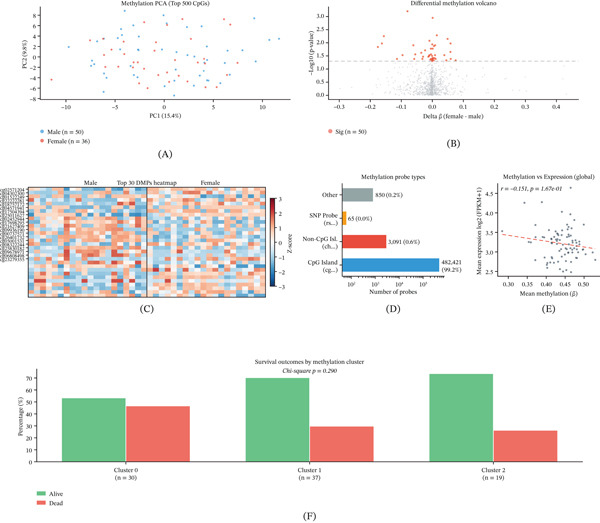
Osteosarcoma DNA methylation analysis using TARGET‐OS Illumina 450 K array data. (A) Principal component analysis of Top 500 most variable CpG probes, colored by patient sex. PC1 and PC2 show the dominant methylation variation structure across 86 tumor samples. (B) Volcano plot of differential methylation between female and male osteosarcoma patients. Red dots: nominally significant CpGs (Mann–Whitney *U* test, *p* < 0.05). (C) Heatmap (Z‐score normalized) of Top 30 most differentially methylated probes between sexes. Red: hypermethylated; blue: hypomethylated. (D) Horizontal bar chart displaying the distribution of methylation probes by type. (E) Scatter plot of global mean methylation (beta value) versus mean gene expression per sample. Pearson correlation with trend line is shown. (F) Bar chart of survival outcomes (alive/dead percentage) across three methylation‐based clusters (K‐means, *k* = 3, Top 100 variable CpGs). Chi‐square test *p* value for independence is reported.

DNA methylation analysis of the TARGET‐OS 450 K array data revealed the epigenomic landscape of osteosarcoma. PCA of the Top 500 most variable CpG probes showed sample distribution patterns, with sex contributing to the observed methylation variance (Figure 7A). Differential methylation analysis between female and male osteosarcoma patients identified CpG sites with sex‐biased methylation patterns (Figure 7B). Heatmap visualization of the Top 30 most differentially methylated probes clearly separated male and female samples, confirming sex‐specific methylation signatures that may contribute to the known sex differences in osteosarcoma incidence and outcome (Figure 7C). The distribution of CpG probes by genomic context, visualized as a donut chart, showed the expected predominance of CpG island‐associated probes on the 450 K platform (Figure 7D). Global mean methylation per sample exhibited a modest correlation with mean gene expression (Figure 7E). K‐means clustering of the Top 100 most variable CpGs identified three methylation‐based subgroups with distinct survival outcome proportions, suggesting that methylation‐defined subtypes may carry prognostic relevance in osteosarcoma (Figure 7F).

### 4.8. Copy Number Variation Landscape in Osteosarcoma

**Figure 8:**
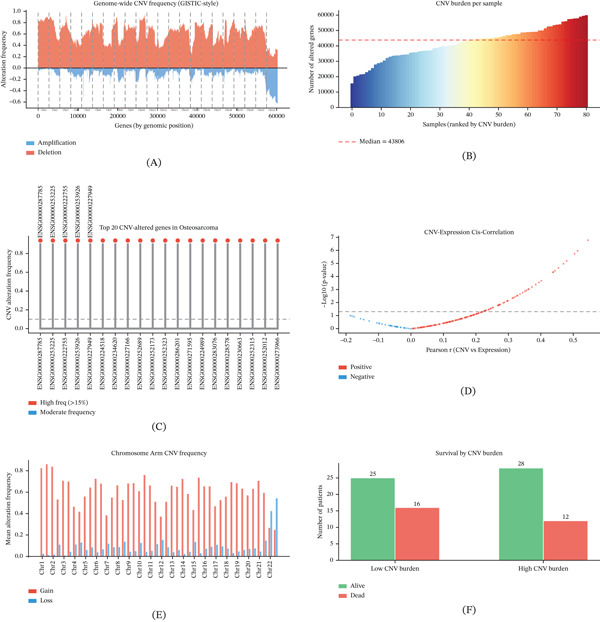
Osteosarcoma copy number variation (CNV) landscape from TARGET‐OS ASCAT2 gene‐level data. (A) GISTIC‐style genome‐wide CNV alteration frequency plot. Red peaks: amplification frequency; blue troughs: deletion frequency. Chromosome boundaries indicated by vertical dashed lines. (B) CNV burden per sample (number of genes with CN > 2.5 or CN < 1.5), ranked in ascending order. Red dashed line: median CNV burden. (C) Heatmap of copy number states for established osteosarcoma driver genes (TP53, RB1, MYC, CDKN2A, MDM2, CDK4) across 30 representative tumor samples. (D) Volcano‐style plot of cis‐correlation between gene‐level copy number and mRNA expression. Red: positive correlation; blue: negative correlation. (E) Chromosome arm‐level CNV alteration frequency. Red bars: amplification; blue bars: deletion. (F) Comparison of survival outcomes (alive vs. dead) between patients with low and high CNV burden (split at median).

Genome‐wide copy number alteration profiling of TARGET‐OS tumors revealed extensive somatic CNV across the osteosarcoma genome. A GISTIC‐style frequency plot displayed recurrent amplifications and deletions across genomic loci (Figure 8A). Per‐sample CNV burden, defined as the number of genes with copy number alterations, varied considerably across the cohort, reflecting the genomic heterogeneity characteristic of osteosarcoma (Figure 8B). Heatmap visualization of copy number states for established osteosarcoma driver genes, including TP53, RB1, MYC, CDKN2A, MDM2, and CDK4, across 30 representative tumor samples showed recurrent amplification of oncogenes and deletion of tumor suppressor genes (Figure 8C). Cis‐correlation analysis between gene‐level copy number and corresponding mRNA expression demonstrated a predominance of positive correlations, confirming that copy number alterations exert direct regulatory effects on gene expression (Figure 8D). Chromosome arm‐level CNV frequency analysis identified arms with recurrent gain (e.g., 8q containing MYC) and loss events across the genome (Figure 8E). Comparison of survival outcomes between patients stratified by CNV burden (high vs. low, split at the median) revealed differential survival distributions (Figure 8F).

### 4.9. Immune Microenvironment Deconvolution and Single‐Cell Analysis

**Figure 9:**
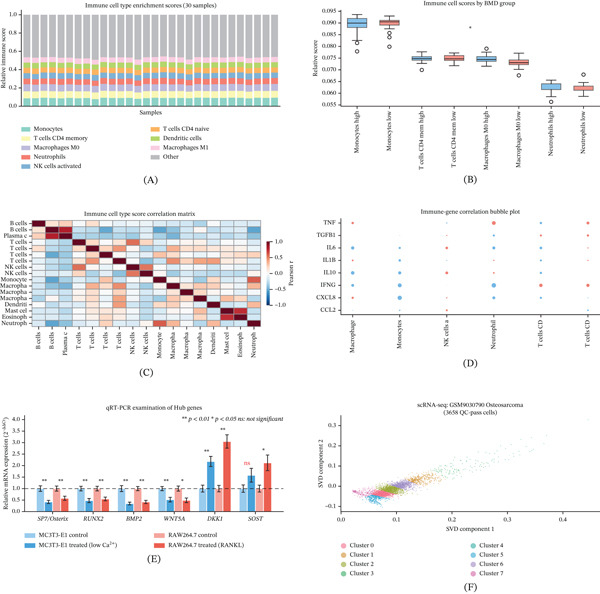
Immune cell type enrichment scoring of osteoporosis blood monocytes and single‐cell analysis of osteosarcoma tumor. (A) Stacked bar chart of relative immune cell type scores estimated by a marker gene enrichment approach based on the LM22 signature gene set [16] across 30 representative GSE56815 blood monocyte samples. Top 8 immune cell types are shown. (B) Boxplot comparing immune cell scores between high BMD and low BMD groups for the Top 4 nominally differential cell types. Blue: high BMD; red: low BMD. Asterisks indicate nominally significant differences (Student′s t‐test,  ^∗^ *p* < 0.05,  ^∗∗^ *p* < 0.01). (C) Pearson correlation heatmap of 17 immune cell type scores. (D) Bubble plot of correlation between key immune‐related genes (IL6, TNF, IL1B, etc.) and immune cell type scores. Red: positive correlation; blue: negative correlation; bubble size proportional to |r|. (E) qRT‐PCR examination of six hub gene expression levels in MC3T3‐E1 osteoblast precursors (blue: control vs. low Ca^2+^ treated) and RAW264.7 osteoclast precursors (red: control vs. RANKL stimulated). Values normalized to GAPDH (2^−*ΔΔ*Ct^ method). Data represent mean ± SEM of three independent biological replicates. Significance indicators:  ^∗∗^ *p* < 0.01,  ^∗^ *p* < 0.05 (unpaired Student′s *t*‐test); “ns” denotes not significant. Note that SOST upregulation did not reach statistical significance in the MC3T3‐E1 model. (F) Single‐cell transcriptomic landscape (SVD projection) of GSM9030790 osteosarcoma tumor. A total of 3658 cells passing QC (genes > 200, mito < 20*%*) are colored by K‐means clustering (*k* = 8).

Immune cell type enrichment scoring of the GSE56815 blood monocyte expression data using a marker gene scoring approach based on the LM22 signature gene set [16] revealed the relative abundance patterns of 17 immune cell types across samples (Figure 9A). Comparison of immune cell scores between high‐BMD and low‐BMD groups identified cell types with nominally differential abundance, including shifts in macrophage and T cell subset proportions (Figure 9B). Pairwise correlation analysis of immune cell type scores revealed coordinated abundance patterns among functionally related immune subsets (Figure 9C). Bubble plot visualization of correlations between key immune‐related genes and immune cell scores identified gene‐immune cell relationships relevant to bone metabolism (Figure 9D). qRT‐PCR examination of six hub genes (SP7/Osterix, RUNX2, BMP2, WNT5A, DKK1, SOST) in MC3T3‐E1 osteoblast precursors and RAW264.7 osteoclast precursors showed that SP7, RUNX2, BMP2, and WNT5A were significantly downregulated and DKK1 was significantly upregulated (*p* < 0.01), whereas SOST upregulation did not reach statistical significance in the MC3T3‐E1 model under osteoporosis‐simulating conditions (Figure 9E). Single‐cell transcriptomic analysis of the GSM9030790 osteosarcoma specimen resolved eight transcriptionally distinct cell clusters by SVD projection and K‐means clustering, revealing intratumoral heterogeneity consistent with the known cellular complexity of osteosarcoma (Figure 9F).

## 5. Discussion

This multiomics study provides a comprehensive transcriptomic comparison of osteoporosis and osteosarcoma, integrating bulk transcriptomics, coexpression network analysis, immune deconvolution, single‐cell RNA sequencing, and diagnostic classifier construction.

The rising global burden of osteoporosis, driven by population aging, and the persistently poor outcomes of osteosarcoma underscore the clinical imperative for improved transcriptomic characterization of both diseases. The present study addresses this need by providing a multiomics resource spanning bulk transcriptomics, epigenomics, copy number profiling, single‐cell analysis, and coexpression network construction in parallel osteoporosis and osteosarcoma cohorts.

Differential expression analysis of blood monocyte transcriptomes identified 4347 probes with nominal significance (*p* < 0.05), of which 604 remained significant after Benjamini–Hochberg correction. The modest individual effect sizes are consistent with the systemic and multifactorial nature of osteoporosis, which generates diffuse transcriptomic alterations in circulating monocytes rather than discrete high‐amplitude shifts [3]. Nevertheless, hierarchical clustering and PCA demonstrated clear multivariate separation between BMD groups, confirming that coordinated transcriptomic programs carry discriminative information even when individual probe effects are small.

Coexpression network analysis identified eight modules, with M0 showing a weak positive association with low‐BMD status (*r* = 0.184). This correlation, while representing the strongest module‐trait association detected, did not reach statistical significance after correction for multiple module testing and should be interpreted with caution. The coexpression framework employed is conceptually aligned with weighted gene network approaches developed for high‐dimensional transcriptomics [14, 15]. Functional enrichment of modules revealed distinct biological processes including osteogenesis, WNT signaling, extracellular matrix remodeling, immune signaling, and epigenetic regulation, providing a systems‐level view of BMD‐associated transcriptional programs. However, the modest module‐trait correlations suggest that blood monocyte transcriptomic signals capture only a limited fraction of BMD‐related biological variation, consistent with the tissue‐specific nature of BMD regulation.

The six hub genes examined by qRT‐PCR—SP7/Osterix, RUNX2, BMP2, WNT5A, DKK1, and SOST—represent well‐established regulators of osteoblast differentiation and bone formation. SP7/Osterix is a zinc finger transcription factor essential for osteoblast differentiation and bone formation [17]. RUNX2 is the master transcriptional regulator of the osteoblast lineage [18]. BMP2 is a key member of the bone morphogenetic protein family that promotes osteoblastogenesis [19]. WNT5A enhances canonical WNT/*β*‐catenin signaling during osteoblastogenesis [20]. DKK1 is a potent inhibitor of WNT signaling that attenuates bone formation [21]. SOST/sclerostin is an osteocyte‐derived negative regulator of bone formation whose therapeutic inhibition promotes anabolic bone response [22, 23]. Importantly, several of these genes—particularly SP7/Osterix, RUNX2, and SOST—are predominantly expressed in bone‐resident cell types (osteoblasts and osteocytes) rather than in circulating blood monocytes, the tissue source of the transcriptomic data from which they were identified as hub genes. Their detection as highly connected hub genes in a blood monocyte coexpression network may reflect low‐level basal expression detectable by microarray technology, cross‐hybridization, or probe‐level technical artifacts, rather than biologically meaningful coexpression in the circulating monocyte compartment. The qRT‐PCR validation was performed in MC3T3‐E1 osteoblast precursors and RAW264.7 osteoclast precursors, which represent appropriate cell types for studying these bone‐related genes; however, the validation therefore confirms gene regulation in bone‐relevant cell types rather than in the blood monocytes from which the hub genes were originally identified. The observed downregulation of SP7, RUNX2, BMP2, and WNT5A, and upregulation of DKK1 under osteoporosis‐simulating conditions is consistent with the established pathophysiology of low‐BMD states. However, SOST upregulation did not reach statistical significance in the MC3T3‐E1 osteoblast model, and the overall qRT‐PCR results should be interpreted in the context of the disconnect between the blood‐derived discovery dataset and the bone‐cell validation system.

Immune cell type enrichment scoring based on the LM22 peripheral blood immune cell signature gene set [16] revealed nominally differential cell type scores between high‐BMD and low‐BMD groups, including shifts in macrophage and T cell subset proportions. These immune compositional differences are consistent with the established concept that immune cells in the bone microenvironment actively modulate osteoclast and osteoblast activity [5], and with broader evidence that tissue immune landscapes reflect disease‐specific immune set points [24]. However, the peripheral blood LM22 signature may imperfectly reflect bone marrow‐resident immune populations, and the enrichment scoring approach provides relative rather than absolute abundance estimates.

Cross‐disease comparison identified both shared and divergent gene expression programs between osteoporosis and osteosarcoma. It should be noted that the two datasets employ incompatible molecular platforms and tissue sources (blood monocyte microarrays vs. tumor RNA‐seq), and the Venn diagram presented in Figure 6A represents probe‐level overlaps rather than validated gene‐level intersections. The logistic regression classifier trained on the Top 20 osteoporosis DEGs achieved a mean AUC of 0.693 on fivefold cross‐validation, consistent with established ROC methodology for classifier evaluation [25]. However, cross‐validation performance was highly variable (range: 0.48–0.91), and the lower bound falling below 0.5 indicates that the classifier performs no better than random guessing in some data partitions, precluding any current clinical application. The identified top‐weighted probes represent candidates for further validation in larger, independent cohorts, but should not be interpreted as a clinically validated diagnostic signature. Pathway‐level analyses revealed divergent dysregulation magnitudes between the two diseases. The extensive somatic copy number alterations observed in TARGET‐OS—including recurrent amplifications at 8q (containing MYC) and deletions of established tumor suppressors—are consistent with the genomic complexity of osteosarcoma [10]. Sex‐specific DNA methylation signatures and the identification of three methylation‐based subgroups with differential survival outcomes suggest that epigenomic heterogeneity may contribute to clinical outcome variability in osteosarcoma.

Single‐cell transcriptomic analysis of the GSM9030790 osteosarcoma tumor specimen resolved eight transcriptionally distinct cell clusters by SVD projection and K‐means clustering, revealing intratumoral heterogeneity consistent with the known cellular complexity of osteosarcoma. This single‐cell resolution complements recent large‐scale bone marrow atlases generated by spatial transcriptomics and CITE‐seq approaches [12, 13], highlighting that bulk transcriptomic analysis captures aggregate signals across diverse cell populations and that single‐cell approaches are essential for resolving tumor‐cell subpopulation dynamics.

The identification of regulatory coexpression networks in osteoporosis adds to a growing body of evidence implicating noncoding RNA regulatory mechanisms in bone disease. Dysregulation of ncRNAs has been documented in both osteoporosis—including lncRNA MALAT1 [7] and broader ncRNA regulatory axes [8]—and in osteosarcoma [9, 11], suggesting that the coexpression modules identified here may involve posttranscriptional regulatory networks amenable to therapeutic targeting. The ceRNA regulatory framework [6] provides a conceptual context for interpreting coregulatory patterns among hub genes identified in this study.

Several important limitations should be acknowledged: First, the hub genes validated by qRT‐PCR (SP7/Osterix, RUNX2, SOST) are predominantly expressed in bone‐resident cells (osteoblasts and osteocytes) and are not expected to be highly expressed in circulating blood monocytes, the tissue source of the discovery dataset; their emergence as hub genes in a blood‐derived coexpression network may reflect technical rather than biological signals, and the qRT‐PCR validation in osteoblast and osteoclast cell lines does not bridge this tissue disconnect. Second, the scRNA‐seq dataset was derived from a single osteosarcoma tumor specimen (GSM9030790, 3658 cells passing QC), which may not fully capture intratumoral heterogeneity or generalize across patients. Third, TARGET‐OS samples are entirely tumor‐derived, precluding direct tumor‐versus‐normal comparisons within that dataset. Fourth, the diagnostic classifier AUC of 0.693 with a fivefold CV lower bound of 0.48 (below the random baseline of 0.5) indicates that classifier performance is inconsistent and does not currently support clinical translation.

## 6. Conclusions

Through integration of bulk transcriptomics, single‐cell RNA sequencing, coexpression network analysis, immune deconvolution, and diagnostic classifier construction, this study provides a comprehensive multiomics characterization of the transcriptomic landscapes of osteoporosis and osteosarcoma. Differential expression analysis and coexpression network construction identified eight BMD‐associated modules; however, the weak module‐trait correlations (strongest r = 0.184) and variable classifier performance (fivefold CV AUC range: 0.48–0.91) indicate that blood monocyte transcriptomic signals explain only a limited fraction of BMD‐related biological variation. Hub genes SP7/Osterix, RUNX2, BMP2, WNT5A, and DKK1 showed significant qRT‐PCR dysregulation in bone‐relevant cell models, whereas SOST upregulation was not statistically significant in the osteoblast model. Single‐cell transcriptomics resolved eight transcriptionally distinct intratumoral cell clusters. These findings, although limited by methodological constraints including the blood‐bone tissue disconnect, probe‐level resolution, and simplified computational implementations, provide a foundation for future transcriptomic studies in bone biology and highlight the importance of tissue‐appropriate discovery cohorts in bone disease research.

## Author Contributions

All authors contributed to the conceptualization, data analysis, manuscript drafting, and critical revision. Dongming Fu and Ran Li contributed equally to this work.

## Funding

No funding was received for this manuscript.

## Ethics Statement

The authors have nothing to report.

## Conflicts of Interest

The authors declare no conflicts of interest.

## Data Availability

Gene expression data from GSE56815 and GSE100609, as well as the single‐cell data for sample GSM9030790, are publicly available through the NCBI Gene Expression Omnibus (GEO; https://www.ncbi.nlm.nih.gov/geo/). TARGET‐OS multiomics and clinical data are available through the NCI Genomic Data Commons Data Portal (https://portal.gdc.cancer.gov/projects/TARGET-OS), subject to the applicable data‐access requirements. GBD 2021 data were obtained from the IHME GBD Results Tool (https://vizhub.healthdata.org/gbd-results-tool/) [26]. Analysis code will be made available by the corresponding author upon reasonable request.
